# Sensor-Enabled Context-Aware and Pro-Active Queue Management Systems in Intelligent Environments

**DOI:** 10.3390/s20205837

**Published:** 2020-10-15

**Authors:** Radosław Klimek

**Affiliations:** Department of Applied Computer Science, AGH University of Science and Technology, Al. Mickiewicza 30, 30-059 Krakow, Poland; rklimek@agh.edu.pl

**Keywords:** queue management system, IoT, intelligent environment, context-awareness

## Abstract

Queue systems are practically used in various institutions and commercial enterprises constituting a challenge for the intelligent environments in smart cities. The management of the flow of customers guarantees the elimination or reduction of the queues as well as the economic benefits which follow the clients’ satisfaction of a better quality of service. An intelligent queue management system has been proposed which is designed as the pro-active and context-aware ecosystem based on multiple low-level sensors and devices constituting the IoT (Internet of Things) network. The designed context-driven system is characterised by user friendliness, as well as the client behaviour recognition and understanding which generate actions that support clients, establishing wealthy environments. A prototype version of the system has been proposed which has been validated by formal analysis and simulation. This prototype can be used as a necessary experience and as a reference point when building a target system and meeting requirements typical for context-aware and pro-active systems based on IoT networks which process massive data streams.

## 1. Introduction

Smart cities which are becoming more and more common and inevitable are understood as places where modern ICT (Information and Communication Technology) technologies are used in order to improve the quality of citizens’ lives. They also enable the sustainable usage of available resources. There is not a single scenario which helps to reach these goals. On the one hand, developing context-driven applications is always hard and complex but the typical problems towards context-aware systems are similar everywhere. The IoT (Internet of Things) technologies have a key role in the development of smart environments [1,2,3].  *Intelligent environments* IEs are spaces realizing ambient intelligence in IoT-based networks with related services [2]. The IoT monitoring requires a means of information exchanging techniques and intelligent reasoning to generate smart decisions. IE aim at seamlessly used computations and services to increase standards of inhabitants’ lives. We can see queues in many places of smart cities, enterprises, as well as various types of clients who behave differently in numerous situations and have distinctive needs or preferences. The main aim of the intelligent queue management system is to adjust to those needs which enable the minimising of the length of queues, shortening the time of customer service and increasing the level of satisfaction with services. Indirectly, it can also influence a growth in the volume of sales.

The aim of this work is to propose an intelligent queue management system for a large store. It allows for the creation of a context-aware and sensor-based system which, in a non-intrusive way, would understand people’s behaviour, categorise them, and manage the queues in a pro-active way. This IoT architecture integrates sensor networks when higher level knowledge is extracted, giving insights that support and optimise the operation within the intelligent environments, increasing the quality of the citizens’ lives. (The queue management system can be implemented not only for needs of the shopping mall but also for objects such as businesses, airports, offices and other places.) The proposed system is based on a multi-agent architecture that guarantees the best tasks dispersion. We have also proposed a contextual data taxonomy, strictly tailored for queue management systems enabling positive contribution to the reasoning processes. The designed system has a conceptual character, but the objective is to prove its technical feasibility and vitality, as well as to establish a wealthy ecosystem. The system prototype can be developed and improved further by introducing new details, as well as different methods of client identification. This prototype can also be used as the reference point when creating the subsequent system, and where its operations of the intelligent environments are optimised, enhancing its efficiency.

The current COVID-19 pandemic times, and the resulting threats have unexpectedly brought forward the potential benefits of such a queue management system. For example, a system deployed over a large area can easily identify people with increased temperature and immediately direct them to the appropriate desks intended for them, thus separating such people. The consequence of this is also the positive influence on the maintenance of the required social distancing. Moreover, many countries have developed applications to track contacts with coronavirus. Thus, combining such an application with our system could give a synergy effect. However, all these threads will not be discussed in this article, as they are out of range at this stage.

The whole article has the following structure. Section 3 discusses the basic issues and fundamental assumptions of the system which consists of system aims of queue management, appearing devices, and their roles. The way of deploying basic-cameras and possible kinds of tills was described as well. The basic use cases and scenarios for clients, served within the monitored area, are presented informally in Section 4. An important proposition is contextual data taxonomy adopted for this system presented in Section 5. This section also contains the basic system architecture. Section 6 includes a proposition of a multi-agent system, its architecture for solving the problem of queue management system. Some implementation details including global variables and messages structures are discussed in Section 7. Section 8 contains the description of all algorithms which are the core for building a basic operational logic of agents. Section 9 includes discussion concerning the proposed algorithms and defined messages. It was performed via the formal analysis of proposed algorithms. Section 10 contains simulation experiments, the results of which were discussed in detail. Section 11 presents the final conclusions and possibilities for further works.

We use the following notations in the article:

- use case diagrams of UML, see [4]—Figure 2,
- workflows and data flow diagrams, see [5,6]—Figures 6 and 9,
- messages as regular expressions, see [7]—Table 2, Algorithms 1–6,
- algorithms expressed as pseudo codes, see [8]—Algorithms 1–6.

The used acronyms are resolved in the end of the article.

## 2. Related Works

There are many works which consider and discuss queue management systems. Moreover, there are also commercial systems for the line management. However, there is a lack of studies regarding smart systems that work in intelligent environments, which is to say systems that understand human behaviour providing context-aware and pro-active actions supporting clients and inhabitants. Such systems are a mandatory component for smart cities. An article by Roy et al. [9] discusses queues in the context of a smart parking system. The aim of the proposed algorithm is to control traffic, avoiding overload when searching for a place to park. Additionally, smart toll tax collection is discussed. A paper by Pandey and Hanchate [10] is another work concerning car parking to avoid unorganized parking and recognizing the parking system as a queue, as well as using queuing theory to suggest a parking lot and avoid peak hours. A paper by Yuan et al. [11] proposes a solution with tags, sectors and algorithms to understand buyer profiles but the contextual data is not considered. However, this is far from our approach where we try to recognise and understand customer behaviour on-line, to support them when queuing. An article by Berdaliyev and James [12] provides methods for queuing delays when using RFID systems. They also use a smart trolleys as an anti-theft system. However, they do not recognise human behaviour. A paper by Stancu et al. [13] also does not discuss behavioural aspects while new message systems are introduced. Some experiences with message brokers are discussed, when considering communication between various data sources and a central application. Some experience with brokers can be used in our work. In an article by Lin and Lin [14], a ticket dispenser is used. It is used to assist customers with the waiting process and support clients with the dynamic prediction of waiting time, when using two different indicators. However, this is far from recognising customer behaviour which is the indispensable aspect of our approach.

Many works can be located in the context, or neighbourhood, of our proposal. They focus on the shopping trolley itself, which can even be automatically counted while shopping. For example, in an article by Chaure and Jain [15] every shopping trolley is equipped with an item identification device to enumerates the total amount of purchasing, an article by Yusuf et al. [16] proposes the smart shopping trolley based on the RFID technology to scan and calculate items in the trolley, an article by Ravindranath et al. [17] proposes also an RFID technology to identify items purchased. A paper by Khairnar and Gawali [18] is another work concernig an RFID for product detections and automatic billing. These articles do not address the queue management problem because they do not refer to queues. On the other hand, they can be used in the future and integrated with our proposal. It would be done in such a way that people approaching the desk indicated by our system would already have a shopping trolley with an added up sum total, which would certainly shorten the service time in the queue itself.

The context definition is provided by the well-known paper by Dey and Abowd [19]. A survey by Augusto et al. [20] investigates the notion from a historical perspective, examining the relationships and interactions between different communities using these notions. A paper by Zimmermann et al. [21] influenced this work by identifying the context categories. We introduced a similar but simpler taxonomy for our system. It is used in reasoning processes. One also encounters the notion of rough sets with context modelling, however, it looks unconvincing since the pre-processed data is rather contaminated with subjective judgments, see the paper by Koczkodaj et al. [22], which reduces the credibility of the input data. A paper by Cheng et al. [23] is a review paper describing researchers’ struggles with the context, but also the context understanding. A paper by Bettini et al. [24] discuss context modelling techniques, introducing levels of abstraction, and requirements which context modelling should meet. Finally, a paper by Dwyer et al. [25] introduces patterns for the system property specification.

Many articles have considered behaviour recognition in ubiquitous computing and urban field is a popular subject of analysis. For example, see a paper by Ratti et al. [26], but most of the work focuses on a stream of behaviours rather than considering an individual one. A paper by Calabrese et al. [27] described mobile phones in an urban monitoring system, based on fixed sensors and GPS receivers and the objective is to visualise vehicular traffic and the movements of pedestrians. Behavioural patterns are also discussed in a paper by Gonzalez et al. [28].

In our system, huge amounts of data are collected by the sensor network, which requires efficient data management. Sensor networks are enablers for IoT, see a paper by Lazarescu [29] or a paper by Mahmud et al. [30], recognized as an effective medium for data collection, but as many new technologies have both barriers and also guarantee synergy. The revolution known as Industry 4.0 is only possible to implement through IoT-based sensor networks and a middleware approach, see a paper by Razzaque et al. [31]. There are many hot topics related to sensor networks. For example, the intrusions detecting based on artificial intelligence, see for example a paper by Otoum et al. [32], where various mechanisms are evaluated, in particular machine learning and deep learning. Intelligent data analytics enable detecting intrusive behaviour in real time, see a paper by Otoum et al. [33]. Another interesting issue is the use of Blockchain technology for managing heterogeneous IoT architecture, see a paper by Dai et al. [34] or a paper by Tseng et al. [35], which is recognised as a promising idea. Interoperability as a property facilitating unrestricted resources’ sharing between various IoT systems is important for our approach. A paper by Carrez et al. [36] provides a reference architecture supporting semantic interoperability for different federated IoTs. This allows for data security during intensive processing typical for IoT systems. Summarizing these works, it should be stated that they are in the circle of our future interest as strengthening mechanisms for the management and security of sensory networks.

The issue of the agentification of the Internet of Things (IoT) was discussed in some research articles; see for example the articles by Maamar et al. [37], and by Kwan et al. [38]. Our approach covers partially the proposed methodology, when considering the definition of an ecosystem, the agentification of things, as well as implementing case studies. Our simulation experiments were influenced by an article by Suryanarayanan et al. [39]. They consider PDES-MAS paradigm and system which is a simulation kernel developed for MAS models supporting when partitioning, load balancing and managing environments in an adaptive manner.

Our work involves the use of facial recognition techniques. Facial recognition software analyses the geometry of a face obtained from a photo or video to create a unique code, in other words a “faceprint”. These techniques are really well developed today, see for example works [40,41,42]. Facial recognition is an important way through which humans interact. It is possible to build a system capable of automatically recognising facial expressions from images and video. It has been an intense field of study in recent years. Researchers have developed methods that can be used to classify and correlate facial expressions. There are many ready-made techniques and systems, see article by Ryan et al. [41]. An article by Corneanu et al. [42] provides a new taxonomy for the facial recognition field. It discusses trends, important questions and the future of research. There are many other types of biometrics which are related to human characteristics, see for example works by Elliott et al. or by Estwood et al. [43,44]. Facial recognition also focuses on expression recognition to extract emotional features, see an article by Zhang and Tjondronegoro [45], or features collected in databases containing variations expected in real conditions, see an article by Zhang et al. [46]. There has been great progress made towards automatic facial expression analysis, see an article by Zhang et al. [47]. Biometric data systems are the rapidly growing area of technology. Biometric data allows the automatic recognition of people attributes based on their distinctive anatomical and behavioural characteristics. For example, physical movements, pregnant women, body temperature, human motion disability, the presence of children, and many others. These problems are not the subject of this paper and will not be discussed here in detail. Similarly we omitted the issues of human privacy in biometric systems, debates on the social and ethical acceptance of biometrics, which are beyond the scope of this paper.

Summarising these works, the dominant observation is that there is a lack of a focus limited to queue intelligent management systems. However, the works have influenced this research by showing a challenging direction for research, as well as considering some patterns of the behaviours, and the categories of the context pieces of data.

Although the idea of the queue management system is not a new one, many of the already existing systems, in spite of claiming to be “intelligent” use mainly the most simple methods of people flow management. They put in place physical barriers or inform users about the overall situation and give statistics related to the already existing queues. Such statistics present only simple information, for example: how many people visited a shop at a particular hour and how much time they spent waiting in queues. However, clients’ on-line identification, rich context situation understanding, and considering present human behaviour with a historical data analysis, combined in assigning clients to particular groups to be serviced in a specific way would be a truly smart and highly desirable solution.

Table 1 summarizes once again all the most important works relating to queue management systems. It should be concluded that these works ignore the broader aspect of smart city operations, namely, a sensor-based system that recognizes and understands customer behaviours, present and past, and through context reasoning provide smart decisions. To the best of our knowledge, our work is the first to address the problem of a queue management system so completely. The proposed system is a prototype and can be treated as a reference point for a potential system of commercial importance. Current business practice does not yet propose such comprehensive smart solutions, and it seems to be a good starting point for further ventures. The basic benefits seem obvious: a significant increase in the comfort of customer service, increased customer safety and increased sales volume. Innovative and socially-accepted products always bring satisfaction for stakeholders.

This work follows up on the paper [48], where the system for intelligent queue management was first proposed. However, the changes and improvements currently made are significant and go much further. The changes include not only a number of clarifications of the system model itself, but also the proposition of a new multi-agent system and its architecture. The algorithmization of such a system is also completely new, previously only the general scenarios of operation were provided, and our algorithms include the operational logic of such a multi-agent system. The queue environment simulator was re-created, which is completely new and has rich functionalities. It allows for the verification of basic assumptions concerning this queue management system, which operates in intelligent environments.

## 3. Basic Assumptions

A *queue* is an impermanent community of people which is created when waiting for a particular event such as being served. The formation of a queue is usually related to the small number of resources/people offering the service to be served. The members of the queue are usually handled as the participants of a set, most often it is the FIFO rule.

The direct objective of the queue system management is to:

- shorten the queues, or to reduce queue service time, balancing the distribution of customers;
- allocate customers to specialised queues and desks in which they will be better served;
- increase customer satisfaction.

We assume that there is a network of devices, which enable the identification of people and events. Among them are:

- basic-cameras, with embedded software which enable recording and provide the biometric data of clients which are in the monitored area; this data allows for initial customer identification (first of all facial recognition), and later this is the basis for in-depth analysis (the later work of the designed system) to recognise the basic characteristics of the clients (elderly person, disabled person, person with children), as well as detecting the increased body temperature, which suggests for example stress, haste, and also illness;
- BLE (Bluetooth Low Energy) sensors: installed in the shopping trolleys and helping to monitor their location together with the client;
- queue cameras located near the cash registers which estimate the number of people waiting in a queue, supported by sensors detecting the users’ Bluetooth and GSM devices to estimate the number of people waiting in a queue (the number of people directed to the queue does not have to be consistent with the number of people present in the queue, which results for example from accompanying persons).

The data types used for contextual analysis and stored in the context warehouse, see Figure 5, can be: age, pregnancy, body posture, weight, clothing, height, body temperature, blood pressure, heart rhythm, amount of shopping. All those data types are gathered and stored in a way which is transparent for a client.

The information generated by the system and messages aimed at clients are introduced to different output devices such as:

- mobile applications which suggest the client a particular behaviour, giving the number of the cash desks where they should go;
- a display on the shopping trolley informing about the number of free cash registers;
- a “help” button on a trolley calling the shop assistant and helping in case of problems with the finalisation of the transaction (elderly people);
- publicly available display at the beginning of each cash register, as well as all other displayers in particular parts of a store.

Devices located in the queue areas are the basis when estimating the length of a queue. However, not only the video cameras are used for this estimation. The Bluetooth devices can also be used, even though this method has its own limits related to the fact that in many mobile phones this function can be switched off. However, it can be useful in describing the time of waiting for each queue, when we assume that at least some people have this function in an active mode, and are observing the movement of the queue.

**Figure 1:**
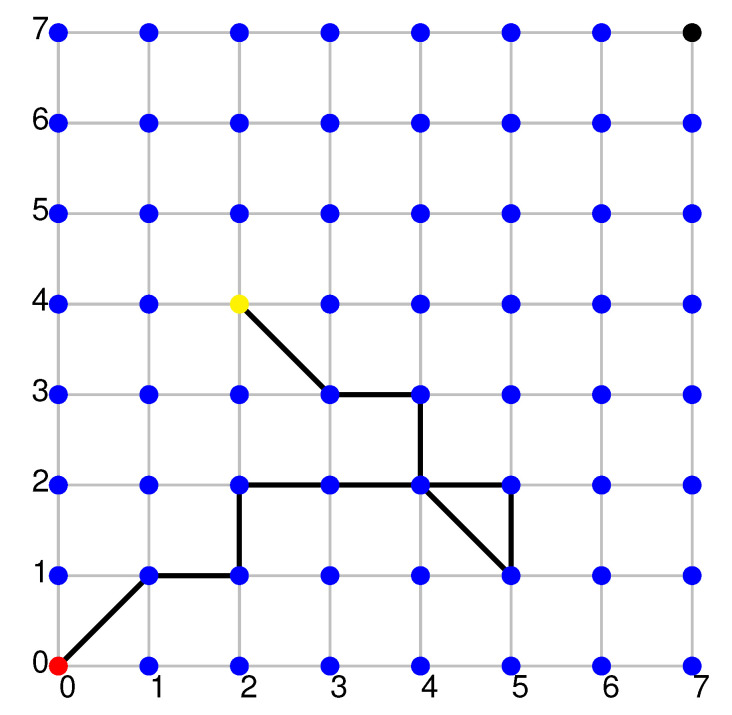
Sample basic-cameras network (Red node—entry, black node—exit, yellow node— current position, visited nodes are connected by edges).

Figure 1 presents an exemplary network of basic-cameras located within the monitored area. The illustration also shows the trace of one sample object, which are nodes where it was observed. The colour red indicates an input node, which is where the object was detected for the first time. The colour black implies an exit area, which is where the tills are located, and thus the node where sooner or later every object will find itself. The colour yellow describes the current position of the object. The edges signify the object’s transitions between the following network nodes.

In general, we could consider a few types of cash registers:

1. special cash desk adjusted to the needs of disabled or elderly people (bigger and wider driveway, bigger displays);
2. cash desks for people with small children;
3. cash desks for people with luxury products who are regular customers;
4. cash desks for the fast service of people with a few products;
5. special cash desks for particular type of products (for example electronics);
6. normal cash registers.

The aim of the whole system, apart from the typical tracing of the clients’ activity and their distance from the cash registers, is also to detect distinctive types of behaviours which enable the redirecting of some people to the cash registers specially designated for their needs. It can be performed on the basis of a historical behaviours analysis, if they are available for the particular clients. This type of system, which is sensitive and context-aware and is characterised by pro-active functioning, aims to increase the level of satisfaction among clients, adjust the sales offers and consequentially increase the trading volume.

After the client identification, we could consider some sample reasoning scenarios as presented below:

1. the person is a regular client—possibility to offer discounts and better standard of service, shorter queues, personal assistants;
2. the increased size of the previous shopping—discounts, redirecting to the cash register for special customers, personal assistants, possibility of shopping delivery;
3. the most frequently chosen categories (household chemistry, groceries, alcohol and others)— information about sales, discounts;
4. buying luxury products—better quality of service;
5. elderly people, pregnant women, people with children, disabled people—special cash desks;
6. when a client moves quickly or slowly, seemingly without a special purpose—people in a hurry are redirected to the shorter queues or the special queues.

## 4. Basic Functionalities

**Figure 2:**
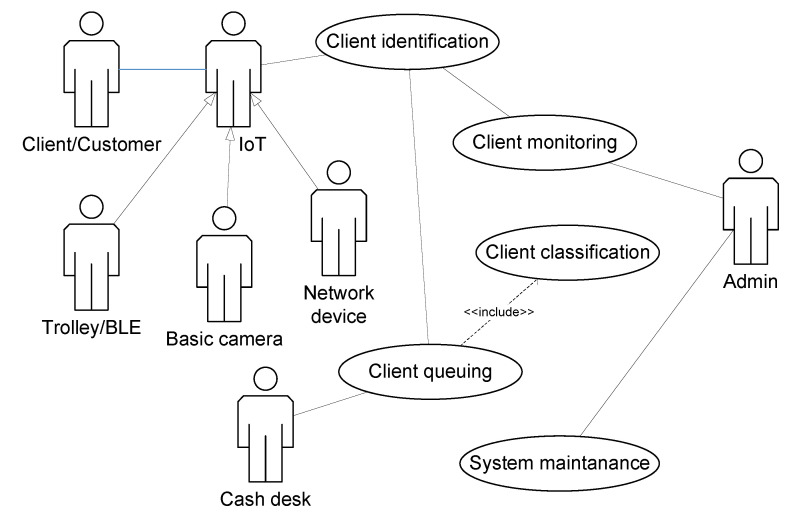
Intelligent queue management system.

Figure 2 shows the basic use case diagram for the intelligent queue management system. *Client* is a single being who stays inside the monitored area and will be served in a queue. The following actors are considered:

- IoT—a physical element (Basic-camera/Trolley-BLE/Network device) which provides the signals from the environment. The information is sent in order to perform a biometric identification. The facial recognition of clients by basic-cameras is of fundamental importance to the system. Client’s physical characteristics (eldery, pregnant, thermal, handicapped, etc.) are provided during later system operations. Some other characteristics (VIP, regular client, etc.) are also supplemented during system operations, see Figure 5;
- Cash desk—subject (a person or a device) providing the customer service for the clients waiting in a queue. Its work should enable the smooth flow of people in a queue and its quick reduction;
- Admin—the physical person who oversees and controls the working of the queue management system, configures its basic parameters and answers the suggestions proposed by the system itself.

The “Client identification” use case is responsible for the biometric identification of a client. The identification is based on facial recognition but it is also supported by other available data emitted by BLE or Wi-Fi network. The “Client queuing” use case provides the final customer service at the cash register such as counting the final amount, updating the information about client in the system, changing the client’s category, if necessary, and all other context information which follows the historic data and which, in the future, will play an important role during the next visit and identification of the client. The “Client classification” use case is included obligatorily in “Client queuing” and it results in at precise analysis of the context data and enables the current classification of the client in order to direct them to the proper cash register. The “Client monitoring” use case is another important part of the system which ensures the current observation of the identified client and the recognition of the moment when directing them to the appropriate cash desk as the place of trade finalisation. Paper [48] provides some general scenarios for selected use cases. The “System maintenance” use case enables the current system management, typical administrative tasks, switching the new devices on and off and deleting the outdated data, keeping it in order etc.

## 5. Context Model and System Architecture

Interpreting the environmental states is of interest for context-aware systems and Ambient intelligence [49]. They are both elements of pervasive computing that bring smartness by playing a key role in the proposed queue managements system.

**Figure 3:**
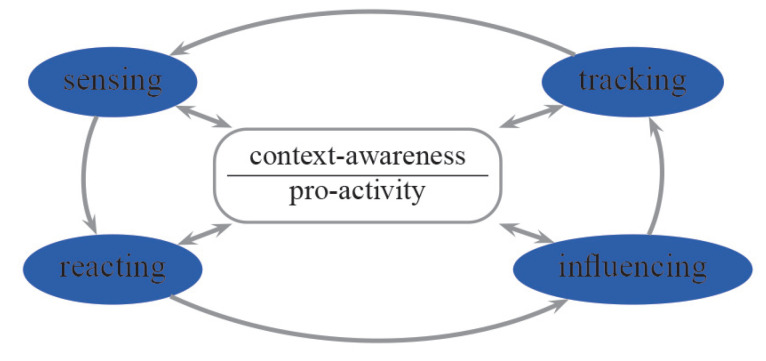
Context-aware and pro-activity systems, see also [48].

The *Ambient intelligence* (AmI) paradigm refers to environments which are sensitive and responsive to the presence of people. The dynamic nature of a typical context-aware system is shown in Figure 3, see also [50], where different phases are repeated periodically, sensing activities and generating proper reactions enabling the operation in a smart environment. The smart environment contains different sensors, or other IoT equipment, distributed in the whole physical area. Smart applications enable understanding a context [21,51], and provide pro-activity, which is acting in advance, in order to to deal with future situations or actions, see [52,53,54].

The idea of context is well established and understood in pervasive computing. The *context* is “...any information that can be used to characterise the situation of an entity. An entity is a person, place, or object that is considered relevant to the interaction between a user and an application, including the user and applications themselves” [19]. Thus, context-awareness is an ability of sensing and reacting to the environment. Sensing and context understanding is necessary and of critical importance for pro-active decisions.

**Figure 4:**
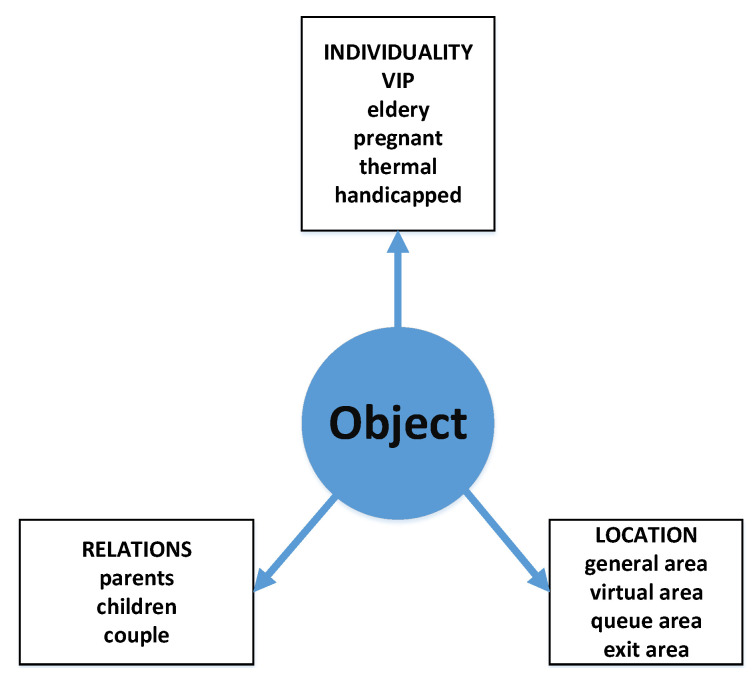
Context model for a queue management system.

**Figure 5:**
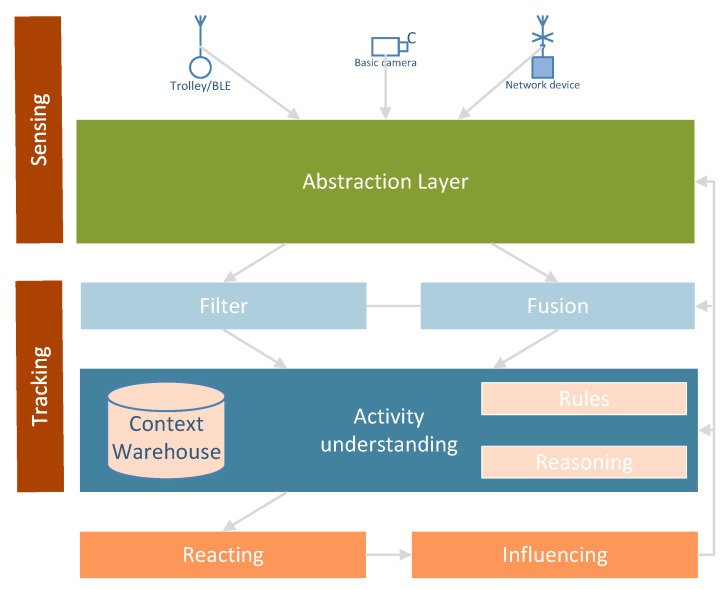
Basic system architecture for activity understanding and context reasoning, see also [48].

Figure 4 shows the proposed context model for the designed queue management system. The adopted model is important for the reasoning processes, see Figure 5. Three basic categories are considered: Individuality, Relations, and Location. At this stage of the system model design, first of all, two categories Individuality and Location are considered. Relations will be included more in future versions of the system.

The overall architecture for the context-aware system that understand clients activities, performing context reasoning processes is shown in Figure 5. The *Context Warehouse* data base captures information engineering, that is:

- to show all the clients attached to the monitored area,
- to provide a consolidated picture of data,
- to capture and provide access to meta data,
- to provide capability for data sharing,
- to merge historical data with current data,
- a deeper understanding of what each clients is,
- how to reconcile different views of the same objects,
- to see if a clients begins behaving uncharacteristically,
- improve the quality of data, etc.

General system architecture operations and rules in Figure 5 are as follows: basic-cameras produce environmental information. Network signals, which are emitted by BLE and Wi-Fi networks, support identifying clients. The *Abstraction Layer* hides some implementation and hardware details. In other words, it translates information between different levels. *Filter* allows the blocking or the removing of some information. *Fusion* enables merging of separate elements into a unified whole. Activity recognition and understanding provide smart decisions, which are to influence the clients in the environments. The *Context Warehouse* is a repository for all information, both historical and present, concerning clients in the environments monitored. *Rules* are how to process and conduct some recognised information. *Reasoning* enables drawing of inferences or conclusions automatically. *Sensing* means gathering and abstracting raw information concerning the monitored environments. *Tracking* is a process of mining information on observed activities. *Reacting* provides smart decisions. *Influencing* implements these decisions in the monitored environment.

## 6. Multi-Agent System

In order to plan the structure of the multi-agent system, we took into account several issues, for example: accessibility, determinism, dynamism, or continuity, see [55]. We will put the emphasis on functional specialization, so that each agent performs strictly defined functionalities, well defined and consistent, and so that the functionalities of any two agents do not have a common part. This approach facilitates future operational decisions by agents, guaranteeing the agents autonomy. Messages are another important issue, we strive to ensure that they are well-defined and that the topology of future message flows are precise and relatively simplified. Some agents exist permanently in the system, but some agents are brought to life and removed during the life of the system; thus, the entire system must be open to such operations. Openess also enables scalability of the system, thai is the creation of new agents from among temporary ones (e.g., GA) does not cause perturbation, also increasing the number of some permanent agents (e.g., QA but also IA) does not change the essence and efficiency of the system operations. Meeting the above requirements allows us to plan the mature agent ecosystem which will be verified later in the paper. Thus, the following taxonomy of agents for the whole system is proposed:

- IA—*Identification Agent*, an agent identifying a client within the monitored area. The identification process can adopt various forms, mainly by the analyses of biometrical data coming from cameras, namely facial recognition, but also via fingerprint readers located on shopping trolleys. IA agent processes the data obtained data and informs GA agent about the new location of the client. IA agent is a permanent part of the system.
- GA—*Guard Agent*, an agent responsible for a particular client who currently remains within the monitored area. It classifies and updates information about the client in the main customer database. It performs the in-depth analysis of the obtained biometric data related to a particular client. The desired result covers attributes such as age, disability, gender, body temperature (which can suggest illness), pregnancy, speed of movement. The GA agent is activated when a client is recognised, for example after entering the monitored area, and deactivated after they leave.
- VA—*Virtual queue Agent*, this agent monitors a virtual queue understood as people moving in the direction of tills but still being hesitant about which one to choose. This agent, on the basis of the data collected, takes a decision about assigning the client to the proper real queue and sends that suggestion to the client. The VA agent is a permanent part of the system.
- QA—*Queue Agent*, manages the specific, real queue which is assigned to a particular till. It has knowledge about the type of queue which is under its control, the number of clients within it, and it estimates the average waiting time, etc. The QA agent is also established permanently in the system and the number of agents is equal to the number of physical queues. After serving a particular client it sends a deactivation signal to their GA agent.
- XA—*Exit Agent*, among its tasks are: observing the fact that clients leave the monitored area and initiating final actions including the deactivation of particular GA agents. XA agent exists permanently in the system.
- MA—*Managing Agent* defines a number of system constants and variables, as well as initialises the system itself. Moreover, it manages the agents of clients who stay within the monitored area.

**Figure 6:**
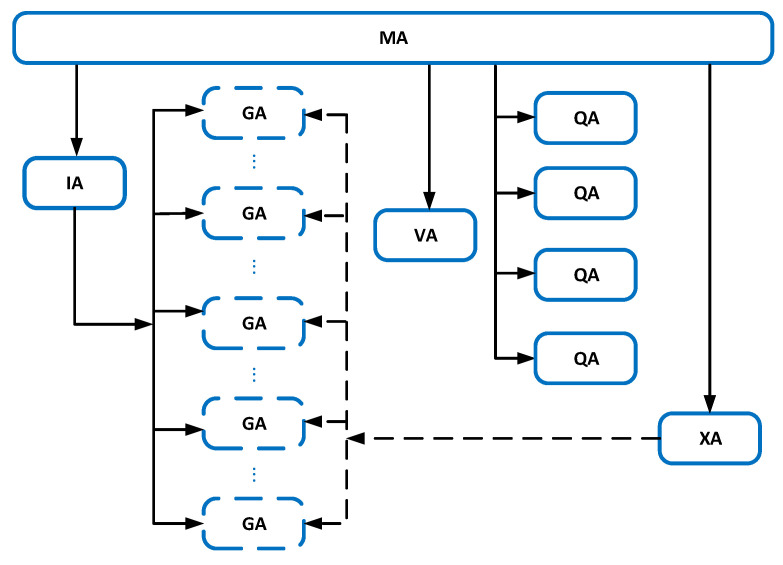
Basic agent architecture and agent relationships. (Solid lines show agents’ constructions and dashed lines show their destructions. Solid ovals show agents which exist permanently and dashed ovals show agents which exist temporarily in the system.).

Figure 6 presents the basic architecture of the proposed multi-agent system. It constitutes a kind of summary but also an illustration for the proposed agent taxonomy. It shows agents’ mutual dependences, both quantitative and qualitative, as well as the agent durations in the system. All agent instances cooperate with each other, exchanging messages, and their operations are oriented to achieve the intended system goals. The algorithmization of agents’ operation will be the subject of a presentation later in the paper, see Section 8, as well as message flows between agents, see Figure 9. The number of GA agents in a system is equal to the number of clients in a monitored area. There is only one VA agent in a monitored area. The number of QA agents is equal to the number of queues within a monitored area. It is assumed that we have four queues now. Although, only one IA agent is presented, it is always possible to increase their number to improve the capacity of the system. Usually IA agent performs a destruction process of GA agents and it is a natural way of removing agents after the client left the monitored area. However, in case of expiry, it can be carried out by MA agent, see Algorithm 1.

## 7. Implementation Details

Although our goal is not a strict system implementation but to obtain a conceptual and prototype version, the most important system variables and messages will be defined in understanding the environment in which MAS operates.

**Figure 7:**
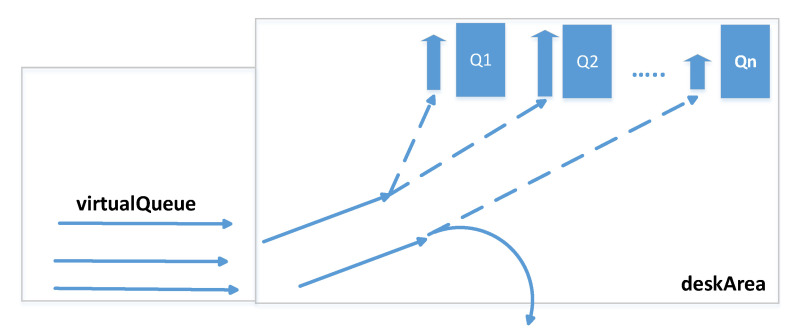
From virtual queue to desk area, or separating clients to queues.

Figure 7 shows an important part of the system which is the organisation of the queue sub-system. The deskArea variable is responsible for the cash desk area which is located close to the physical cash registers (both serviced and those which require self-service), see also [48]. It is supposed that every client who enters this area wants to finish their shopping and is moving closer to the physical cash registers. This fact is detected immediately by the system and such a client is added to the virtualQueue queue. It is not the physical queue but certain information in the system which means that clients are reaching the physical queue area, that is to say they move closer and closer to the queues, and it is necessary to start the queueing reasoning processes. One of the processes is checking the virtualQueue constantly and after the clients classification and the reasoning process, see Figure 5, also making use of the historical data, it chooses the target queue for a client, for example: the queue for elderly people or for people in a hurry. The client, after choosing the queue, is monitored and serviced by the system (mobile phone, trolley displayer or the widely available displayers within the range of the system) which informs them about the target queue. If the client joins the physical queue, the information is removed from virtualQueue. If suddenly the client leaves the deskArea, for example resigns from transaction finalization, they are also removed from the queue sub-system, that is from the virtualQueue and deskArea.

Finally, we decided to consider the following four queue categories:

1. VIP,
2. thermal,
3. special (handicapped, pregnant, or elderly),
4. ordinary/normal.

**Figure 8:**
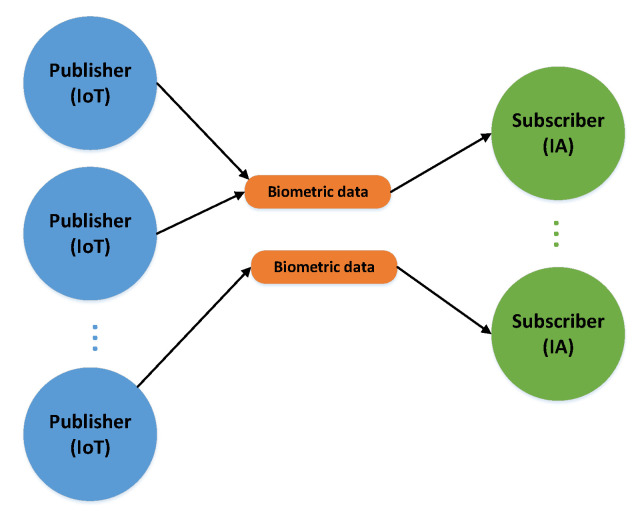
Structure of topics for publisher-subscriber mechanism (a fragment).

The message broker system plays an important role in our system. It is responsible for transporting information, first of all, from basic-cameras, see Figure 1, to agents processing this information. We decided to use Kafka [56,57] platform. It is an efficient platform, which enables the mass sending of different types of data streams. Our experiments were limited to the pieces of data from basic-cameras. Figure 8 presents initiating topics in the system. Messages from each camera are sent to the IA agent, which analyses them in terms of customer recognition in the monitored area. Kafka proved itself to be an efficient medium for the transmission of huge message streams, as was already shown in the paper [54] (Section VI.B), also when the particular system elements were dispersed in a computing cloud [54] (Figure 6), it mirrors the real conditions very well.

In addition to Figure 1, we define the following areas for all basic-cameras as a predefined and enumerated types customerServiceAreas=(generalA,virtualA,queueA,exitA). They mean, respectively: general area, virtual queue area, physical queue area and exit area. Each basic-camera can belong only to one of these four areas.

A list of more important variables in the system includes:

- client=(obId,timeStamp,iotId,deskId)—a list of clients currently staying in the monitored area, where id is the unique customer identifier, timeStamp is the time moment the client was last observed, iotId is the unique IoT/basic-camera identifier where the client was recently observed and $iotId^{\prime }area\in customerServiceAreas$, and deskId is the desk identifier where the client is to be serviced;
- undefBioObs—this variable has an auxiliary meaning and is only used to count the situation when the object could not be identified biometrically. Indirectly, this is a measure of the effectiveness of the system, and possibly a hint on improving biometric identification methods in the future.

Moreover, the structure of the most important messages in the system is shown in Table 2.

## 8. Algorithms

Below we have presented the basic algorithms which create the fundamentals for the proposed multi-agent system, see Algorithms 1–6.

Algorithm 1 illustrates initiation of basic system variables as well as starting the operations of particular system agents. The algorithm does not possess any input or output data in the classic sense. We assume that the system is able to initiate itself and that the final report of its activities is not generated so as not to complicate the algorithms. Certain variables influencing its work can be modified by an administrator.
**Algorithm 1** The MA agent operations (MA-operations).  1: clients:= ∅, undefBioObs:= 0; 2: **start agent**
IA—operations, VA—operations, XA—operations; 3: **start agent** all QA—operations according to the categories    
▹ i.e.: VIP, thermal, special, normal 4: Set auxiliary variables d1, d2, d3, *…* 5: Initialize a message broker system;                          ▹ see Figure 8 6: Set other system parameters.

Algorithm 2 performs the primary identification of clients within the monitored area. It obtains data from all basic-cameras. (In this paper, in order to simplify the analyses, the existence of one IA agent is assumed; however, there are no obstacles to introduce more clone agents performing identical services which can boost the effectiveness and speed of the system, if necessary. The increased number of agents can operate the message broker system successfully.) The function objectRecognition, see line 6, performs the basic recognition of a client. This recognition is based on facial recognition, which is understandable and common today, and other methods of identification may be introduced in the future. If it is a new client within the monitored area, its personal agent is initiated, see line 9. Depending from where the client was first noticed, the message is sent, see lines 12–22, to the proper agent, that is VA or XA.
**Algorithm 2** The IA agent operations (IA-operations).**Input:** reading: bioObs;                         ▹ new biometric observation**Output:** ob: obId, timeStamp, iotId;                       ▹ when construct GA**Output:** reading:bioObs;                           ▹ when sent to GA**Output:** ob: obId;                                ▹ when sent to VA, XA  1:**loop**  2:    get(reading);  3:    **if** reading=nil **then** delay(d1)                    ▹d1 established by admin  4:    **else**  5:        **if** reading.bio≠nil **then**                ▹ else bio is undefined, not recognized  6:           ob:= objectRecognition(reading.bio);        ▹ data in ContextWarehouse, see Figure 5  7:           **if** ob∉clients **then**                      ▹ new agent GA is necessary  8:               cli:= (ob,reading.timeStamp,reading.iotId,null);     ▹ null means not yet selected  9:               **start** GA—operations(ob,cli);                         ▹ construct  10:           **end if**  11:           send(GA’ob,reading);              ▹ to analyse data in a more detailed way  12:           **if** $reading.iotId^{\prime }area$=virtualA **then**  13:               send(VA,ob)  14:           **else**  15:               **if** $reading.iotId^{\prime }area$=queueA **then**  16:                   send(VA,ob)  17:               **else**  18:                   **if** $reading.iotId^{\prime }area$=exitA **then**  19:                       send(XA,ob)  20:                   **end if**  21:               **end if**  22:           **end if**  23:        **else**  24:           undefBioObs++                    ▹ unsuccessful, auxiliary counting  25:        **end if**  26:    **end if**  27:**end loop**

Algorithm 3 shows the way the agent operates for an observed client. Every such client has its own agent. The global variable clients is updated. Every new piece of data obtained from the system observations is recorded in the system database ContextWarehouse, see line 12, to update the client’s trajectory. The collected basic bio data is a subject of a deeper analysis, see line 13, both the current and historical records. This is the right moment for a deeper analysis of centextual data (pregnant, handicapped, children, etc.) for a client and particular GA agents are responsible for these operations. In this way it is also possible, for example, to learn that a currently observed client made a large and luxurious purchase which enables classifying them as the VIP category or that a person was using crutches before and has a physical disability which enables classification of them to the special group. This type of analysis is not the subject of the detailed considerations in this article and the rules of conduct are connected with accepted *Rules* and *Reasoning* components, see Figure 5.
**Algorithm 3** The GA agent operations (GA-operations).**Input:** reading:bioObs;                    ▹ biometric observations for object id  1:**constructor** GA  2:   **critical** clients:=∪{(obId,timeStamp,iotId,null)} **end**               ▹ new client  3:**end constructor**  4:**destructor** GA  5:   **critical** $clients:=\setminus \{\left(GA^{\prime }obId,*,*,*\right)\}$ **end**            ▹ remove client (star means any value)  6:**end destructor**  7:**loop**  8:    get(reading);  9:    **if** reading=nil **then** delay(d2)                       ▹d2 established by admin  10:    **else**          ▹ updating both clients (global var) and ContextWarehouse (see Figure 5)  11:        update clients with GA’obId and reading.timeStamp and reading.iotId;  12:        store reading.timeStamp and reading.iotId to update the trajectory of GA’obId;  13:        analyse reading.bio to obtain more contextual data for GA’obId  14:    **end if**  15:**end loop**

**Algorithm 4** The VA agent operations (VA-operations).
**Input:** ob: obId;                                        ▹ object**Output:** ob: obId, desk: deskId;                            ▹ object and desk  1:
**loop**
  2:    get(ob);  3:    **if** ob=nil **then** delay(d3)                         ▹d3 established by admin  4:    **else**  5:        **if** $ob.iotId^{\prime }area$=virtualA **then**  6:           desk:= reasoning(ob);                             ▹ see Figure 5  7:           clients[ob].deskId:= desk;  8:           broadcasting(ob,desk)  9:        **else**  10:           send($QA^{\prime }desk$,ob)  11:        **end if**  12:    **end if**  13:
**end loop**



Algorithm 4 performs the selection of a queue for a client moving in the direction of a queue zone, so the key factor in this case is the reasoning process, see line 6. Reasoning is conducted on the basis of all the gathered pieces of information related to the particular object ob. The current trajectory of movement across the monitored area as well as biometrical readings are analysed, taking into consideration historical data as well. For instance, the current readings could detect higher body temperature of a client, the historical data that they belong to the VIP group. The higher temperature can prejudge sending this person to a special queue. In the reasoning process the special meaning has prepared rules, see Figure 5. After choosing a till this information is sent (broadcasting) to all accessible devices, both the client’s private ones as well those widely available, that are publicly accessible within the monitored area.

Algorithm 5 demonstrates operations connected with customer service in a particular queue. There are as many QA agents as there are physical queues. The customer service procedures are omitted here not to complicate the algorithm. The main aim of this system is bringing the client to the physical queue. The procedures cover continuous client observations, for example using special additional cameras and wi-fi routers, customer service together with their movement to the exit area.
**Algorithm 5** The QA agent operations (QA-operations).**Input:** ob: obId;                                       ▹ object  1:**loop**  2:    get(ob);  3:    **if** ob=nil **then** delay(d4)                      ▹d4 established by admin  4:    **else**  5:        normal clients’ observation and service;  6:    **end if**  7:**end loop**

**Algorithm 6** The XA agent operations (XA-operations).
**Input:** ob:obId;                                      ▹ object  1:
**procedure** timeOut
  2:    **loop**  3:        **for** ∀c∈clients **do**  4:           **if** $(\left(c.iot^{\prime }area=generalA\right)$ **and** (currentTime−c.timeStamp>time2general)) **or**  5:               $((c.iot^{\prime }area\in \left(virtualA,queueA\right)$ **and** (currentTime−c.timeStamp>time2desk)) **then**  6:               **destruct** $GA—operations^{\prime }c.id$              ▹ expired overly long stay: agent GA, destruct  7:               ogranizeReadings(c.id);               ▹ in ContextWarehouse, see Figure 5, if necessary  8:               updateRules(c.id)                               ▹ see Figure 5, if necessary  9:           **end if**  10:        **end for**  11:        delay(d5)                                       ▹d5 established by admin  12:    **end loop**  13:
**end procedure**
  14:**start process** timeOut;  15:
**loop**
  16:    get(ob);  17:    **if** ob=nil **then** delay(d6)                              ▹d6 established by admin  18:    **else**  19:        **destruct** $GA—operations^{\prime }ob$                            ▹ normal exit, agent GA, destruct  20:        ogranizeReadings(ob);                     ▹ in ContextWarehouse, see Figure 5, if necessary  21:        updateRules(ob)                                  ▹ see Figure 5, if necessary  22:    **end if**  23:
**end loop**



Algorithm 6 is connected with leaving the monitored area by clients staying there, namely with moving to the exit area. The collected data, concerning the object staying in the monitored area, is organised. The organisation process is related, firstly, see line 20, to trajectory by object ob, and also to the detected object features, which is obtained from the following biometrical data that, in the future, during subsequent visits, can be used (size of shopping, higher body temperature, physical disability). In rarer cases the update can be related to the reasoning rules, see line 21, for example, with the simultaneous increase of the volume of purchases by all clients, raising the threshold for classifying clients as VIPs. On the other hand, process timeOut controls those clients who stay too long within the monitored area or if their leaving moment was not captured. It takes place via comparing the current system time (currentTime) with times of the last successful biometric observations of particular clients. As mentioned before, it can happen that a client has already left the monitored area but it was not observed in the exit area until after crossing the till zone, for example if the facial recognition failed. Instruction in line 4 searches for clients staying too long (time2general) in the basic area (generalA), or too long (time2desk) in the till area (virtualA, queueA). In that case, the agents related to those clients are removed from the system. Thus, there are two basic calls for agent destruction in the GA system.

The presented algorithms for agents’ operations provide the basic knowledge regarding the algorithmization of the whole problem. That was one of the goals of this paper, to show that by basing it on a sensory network with massive data flows we can design an intelligent queue management system.

## 9. Evaluation of Algorithms

Examining *termination problem*, which is a description of whether the program will finish running, and finding *time complexity*, which is the total time required by algorithms to run, are important for any algorithm. Please see the following statements below:

Algorithm 1 always terminates. Proof: The algorithm does not contain recursions. All instructions are defined precisely.

Algorithm 1 has Θ(1) complexity. Proof: All instructions are precisely defined and have constant costs.

Algorithm 2 always terminates. Proof: The algorithm does not contain recursions. All instructions are defined precisely. The infinite loop symbolises the readiness for constant processing and can be changed, when necessary, into a loop with an exit condition.

Algorithm 2 has Θ(c) complexity, where *c* is the total number of observed clients. Proof: The algorithm has a dominant loop running through *c* clients, observed as visitors in the entire destination. Readings can be repeated many times, but always in relation to the observed number of clients. Finally, the average value is Θ(c).

Algorithm 3 always terminates. Proof: The algorithm does not contain recursions. All instructions are defined precisely. The infinite loop symbolises the readiness for constant processing.

Algorithm 3 has Θ(r) complexity, where *r* is the total number of client’s readings. Proof: The algorithm has a dominant loop running through *r* readings for a particular client. Readings can be repeated many times. Finally, the average value is Θ(r).

Algorithm 4 always terminates. Proof: The algorithm does not contain recursions. All instructions are defined precisely. The infinite loop symbolises the readiness for constant processing.

Algorithm 4 has Θ(c) complexity, where *c* is the total number of observed clients. Proof: The algorithm has a dominant loop running through *c* clients which approach the desk area. Finally, the average value is Θ(c).

Algorithm 5 always terminates. Proof: The algorithm does not contain recursions. All instructions are defined precisely. The infinite loop symbolises the readiness for constant processing.

Algorithm 5 has Θ(q) complexity, where *q* is the total number of observed clients in a particular queue. Proof: The algorithm has a dominant loop running through *q* clients which are served in a particular queue. Finally, the average value is Θ(q).

Algorithm 6 always terminates. Proof: The algorithm does not contain recursions. All instructions are defined precisely. The infinite loops symbolise the readiness for constant processing.

Algorithm 6 has Θ(c) complexity, where *c* is the total number of observed clients. Proof: The algorithm has two dominant loops running through both clients which approach the desk area, and clients which were lost (omitted, facial observations failed, etc.) These two groups together give the number *c*. Finally, the average value is Θ(c).

The entire system, understood as a group of cooperating agents and their Algorithms 1–6, has Θ(c·r) complexity, where *c* is a number of clients in the monitored area and *r* is a number of readings from basic-cameras, that is sensors, for clients, informally, the frequency of such readings. Proof: We use the parenthesis structure to show the calling of algorithms in the entire system:

A1((…,A2,A4,A6,…,A5,A5,A5,A5,…)·A3,⋯,A3)

where letter “*A*” refers to the particular algorithm and the bottom dots specify other/normal instructions outside the calling point, and the middle dots specify multiple instances. The *A*3 algorithm works concurrently with other algorithms, receiving and processing data from them, so we have introduced additional parentheses. Thus, using the previous statements, we get the following results. *A*2 has costs Θ(c). *A*4 has also costs Θ(c). *A*6 has also costs Θ(c). Every *A*5 has costs Θ(q), but considering all *A*5 together we obtain Θ(c). Every *A*3 has costs Θ(r). Thus, we obtain A1((…,Θ(c),Θ(c),Θ(c),…,Θ(q),Θ(q),Θ(q),Θ(q),…)·Θ(r),⋯,Θ(r)), or equivalently A1((…,Θ(c),Θ(c),Θ(c),…,Θ(c),…)·Θ(r)). A1 provides Θ(1). Finally, the complexity of the proposed system is Θ(c·r).

**Figure 9:**
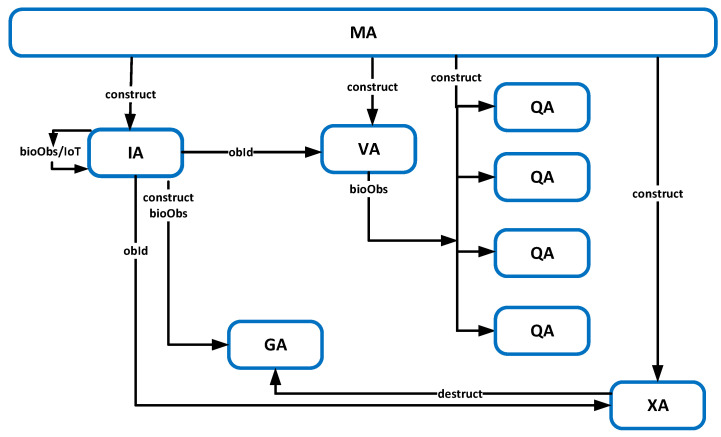
Basic data flows in the system. (Only single instance of GA process is shown.).

Figure 9 shows basic data and message flows in the system, providing a form of system verification. The entire system is initiated with the following basic set of agents: MA, IA, VA, QAs, and XA. When no customer is recognised, then no GA agent is created. All the demonstrated flows are consistent with the proposed algorithms, see agents’ constructions, destructions and normal calls.

## 10. System Simulation

The simulation environment has been created, or more precisely, the prototype of an application, which simulates the work of the intelligent queue management system. Even if the prototype has been simplified, it still portrays precisely how the system works. Moreover, it is much more accurate compared to the environment and experiments presented in article [48]. After the basic operational logic of agents was implemented for the proposed system, the next step was to perform the simulation of the system in order to verify the basic assumptions as well as to explore the possibilities and viability of the queue management system together with the analysis of context data coming from sensory networks.

The time of the whole simulation process is expressed in seconds as an input parameter. Another input parameter is a simulation mode which results directly from the frequency of the occurrence of new clients within the monitored area. The six following simulation modes, characterised by time intervals expressed in seconds for a new client to appear in the monitored area, are distinguished as:

1 very low—[50,800] s,
2 low—[30,600] s,
3 medium—[30,180] s,
4 high—[10,100] s,
5 very high—[10,60] s,
6 ultimate—[1,10] s.

Thus, the interval [a,b] means that the period of time *t*, after which a new client will appear in relation to the previous one, is expressed as a<t<b. The next parameter of simulation process is a size of area where clients stay, the area is understood as a set of basic-cameras monitoring clients’ movements within the shopping area, see Figure 1.

For the purpose of the simulation process it was assumed that the queues have their priorities and are compatible, from the highest to the lowest, with the following list: VIP, thermal, special and normal. If a client could be assigned to two, three or even all of the queues, the one with the highest priority, will be pointed out. On the other hand, it is possible to modify the imputation mechanism in the future by changing the operational logic of the VA agent. For the needs of the simulation, one for each queue category was defined, see Listing 1.

During the simulation process the knowledge about clients, staying within the monitored area, is updated on an ongoing basis. The knowledge consists of: paths made up from nodes where clients were moving around, being the effect of the analyses of data coming from cameras, the times of transitions between the particular nodes, the basic knowledge about all (four) active queues, information about clients adjusted to the particular queues, etc. Collected simulation logs provide us with necessary information about different statistics, for instance: an average queue waiting time, number of pregnant women, number of physically disabled people, etc.

At the very beginning of the simulation process, the pool of clients, together with their historical data (VIP, the size of previous shopping, the volume of purchased luxury items, pregnant women, etc.) are generated. Some of them, possibly even all of them, will take part in the simulation process, if only it takes long enough. The next step is creating physical queues in the system (QA agents). After creating the system environment, the proper stage of simulation starts. Every client appearing in the system is identified by IA agent. Biometrical data in the system is represented in the clients database by a unique hash abbreviation, see Figure 14. Every client receives their own GA agent. If a client appears in the area of tills, it is observed by virtual queue agent VA which role is to decide about further allocation of the client on the basis of the personal data collected (present and historical). The physical queue agent QA adds to, or removes from, the queue client and manages its state. When the client moves towards the till, his/her agent is removed from the system. The flow of the whole simulation process is saved in the system’s logs.

The monitored area is represented by a basic-camera network, see Figure 1. The images from every camera are sent by a message broker to the IA agent.

The explanation of the way of moving for object/client in the monitored area is informally presented in the following algorithm created for the purposes of the simulation process:

1. A selected new client appears at the entry node, see Figure 1;
2. The length of his/her future way of moving is estimated, that is the number of edges in the graph;
3. The time of moving from every *i* node to i+1 node is estimated. When the time passes by, the client moves to a random neighbouring node with a new random time of moving, etc. Those actions are repeated as long as the full length of initially presumed path will be covered entirely;
4. If a final node of the path corresponds to the node of a virtual queue, we can skip to the next one, in this case the last point of this algorithm. If the final node does not correspond to the node of a virtual queue, the shortest path leading from the current point towards the virtual queue is chosen randomly. (Choosing this additional path corresponds to the situation when a client during walking around the monitored area realised that there was an urgent necessity to leave the shop and finalised shopping in one of the tills);
5. For a client who stays in the virtual queue the normal operations connected with choosing and suggesting to them a physical till close to the exit are taken.

The informal algorithm presented above always terminates, and its computational complexity depends on the number of selected edges to pass through, plus possible additional edges finalising client’s duration of stay. On the other hand, the current and exemplary situation around the tills during the simulation process can be observed thanks to logs implemented on a console, for example see Listing 2.

**Figure 10:**
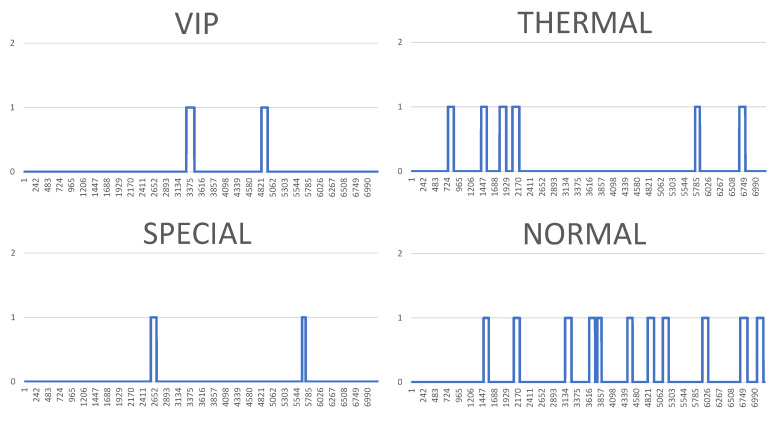
Results of the simulation processes carried out, or queue loads over the simulation time—low mode. (Horizontal axes show the passing of simulation time from 0 to approx. 7000 s, vertical axes illustrate the current number of clients in particular queues.).

**Figure 11:**
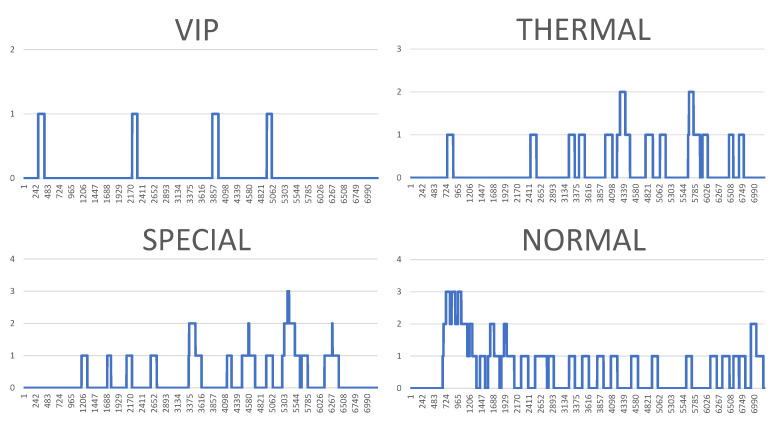
Results of the simulation processes carried out, or queue loads over the simulation time—medium mode. (Horizontal axes show the passing of simulation time from 0 to approx. 7000 s, vertical axes illustrate the current number of clients in particular queues.).

**Figure 12:**
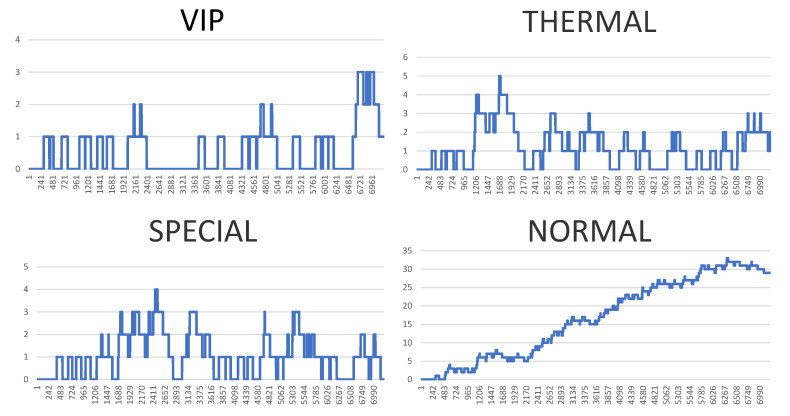
Results of the simulation processes carried out, or queue loads over the simulation time—very high mode. (Horizontal axes show the passing of simulation time from 0 to approx. 7000 s, vertical axes illustrate the current number of clients in particular queues.).

**Figure 13:**
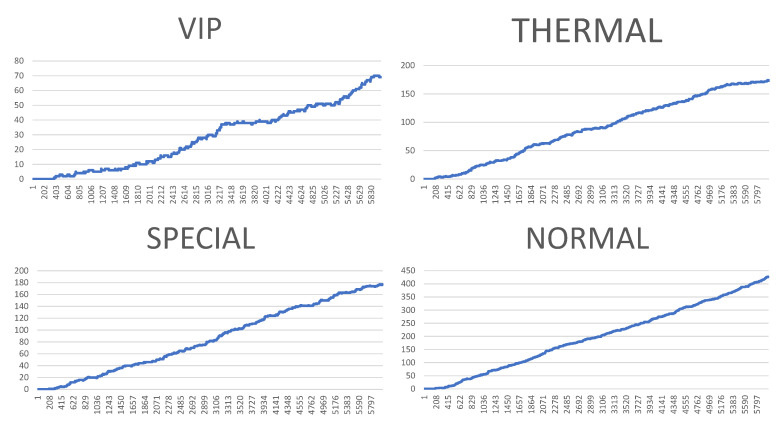
Results of the simulation processes carried out, or queue loads over the simulation time—ultimate mode. (Horizontal axes show the passing of simulation time from 0 to approx. 7000 s, vertical axes illustrate the current number of clients in particular queues.).

However, the visualisation of both system logs as well as the visualisation of the whole generated simulation reports is also possible. Figure 10, Figure 11, Figure 12 and Figure 13 illustrate some of obtained simulation results, namely, the results of four out of six simulations. Every diagram presents a load of particular queues in the passing of simulation time. Figures correspond to the following simulation modes: “low”, “medium”, “very high”, and  “ultimate”, respectively. Every figure contains the following queues: VIP, thermal, special and normal. The growing queue load in relation to subsequent simulation modes is clearly visible. It is already high in case of the “medium” mode. In case of the “very high” mode the normal queue is overloaded. According to the simulation’s initial assumptions, there is only one queue of every type in the system, see Listing 1, which, in a normal life situation, would rather require running other equivalent desks for the queue of a normal type in order to defuse the overload and improve service comfort for the clients. Also, in case of the special queue, there is a possibility to open a new till and desk temporarily. In case of an “ultimate” mode all tills are overloaded definitely, which  means mobilising additional desks for every type of till. Another issue is the fact that this simulation mode is extreme in its assumptions in relation to market newcomers. In practice, the “ultimate” mode should not take place and its presentation here had only an experimental character. In other words: under such an ultimate load of clients it is very difficult to talk about an effective client management since new clients are appearing in the monitored area every few seconds or even every second. On the other hand, it should be noted that the whole simulation presented above, including the “ultimate” mode, demonstrated the service feasibility in the case of the designed system, as well as the authenticity of the system’s basic assumptions.

**Listing 1:** Defining queues.

# queue_type: count, there must be
# at least one queue for each type
queue_config={
QueueType.VIP: 1;
QueueType.THERMAL: 1;
QueueType.SPECIAL: 1;
QueueType.NORMAL: 1;

**Listing 2:** An example of queue status. Each asterisk represents one client.

Time: 501 [s]
vip: *
thermal: *
special: **
normal: ****

**Figure 14:**
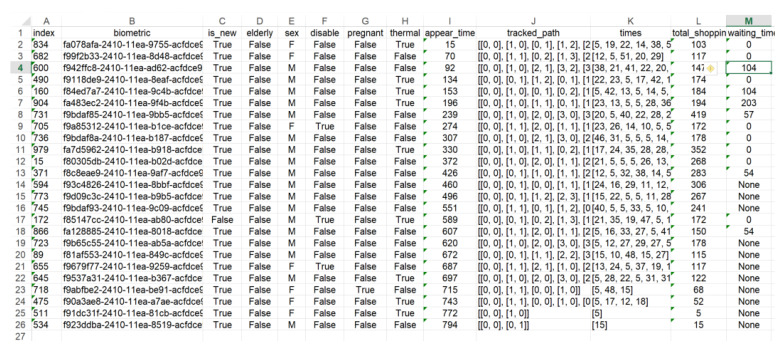
Basic simulation data related to clients.

Figure 14 presents one of the basic reports from the simulation process. It is created separately for every of six simulation modes. Most of the data included in the table is easily understandable. However, some explanations are necessary, for example, row J, contains a path of a particular client within the monitored area. Column K shows the times of passing through to the following nodes and it is comprehensible that the number of figures corresponding to times is always one lower than the number of nodes. For example, 20-th row provides the path ((0, 0), (0, 1), (1, 1), (2, 2), (3, 2), (3, 3)) and partial times (15, 10, 48, 15, 27). This client has not reached the queue area yet. Column L demonstrates the entire shopping time and column M presents the waiting time in the queue. The null value corresponds to an empty queue, which means the client was served without waiting. Value “none” means that the client has not reached the queue yet, there is still in the shopping process.

**Figure 15:**
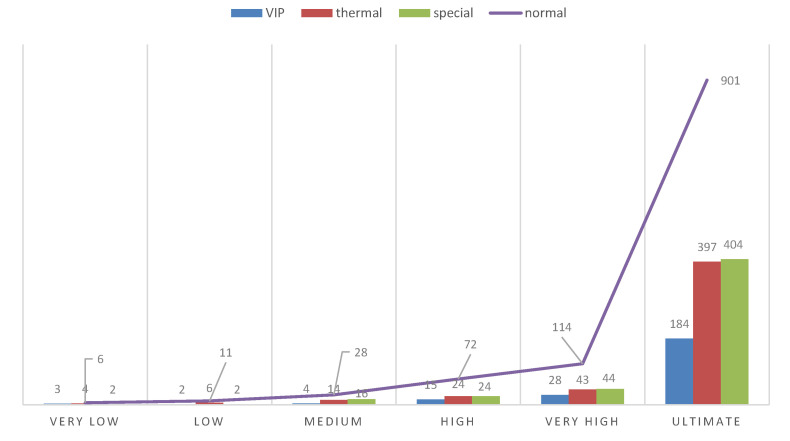
Six simulation modes. (The numbers mean the number of people handled in the particular types of queues plus people who were in the queues at the time of the simulation interruption.).

Figure 15 shows a summary table of all six simulation modes. One simulation process illustrates processes of real world for more than 7000 s, which is almost 2 real hours. (For obvious reasons the simulation itself takes much less time.) The numbers next to each bar mean the number of people handled in the particular types of queues plus people who were in the queues at the time when the simulation was interrupted. It needs to be emphasised that in each and every simulation mode we were dealing with only four desks, one for each type of queue. In a real situation it would be necessary to increase periodically the number of particular desks according to demands. To sum up, the following conclusions can be made. Those six simulation modes cover a wide range of behaviours and situations, from a case when there are only a few clients to one where there is a very high number of clients and they appear every few seconds. It requires further analysis if those modes are to match real-life situations. However, our goal was the testing of principles of the proposed and designed multi-agent system, coming from a wide spectrum of load possibilities. In this sense the experiments can be considered as successful and proving the feasibility and viability of the designed system. On the other hand, none of those particular simulations, taken as a single unit, can be responsible for the entire daily working time of a monitored area because there are periods when the intensity of the clients’ flow is very diverse. However, it is a topic for further research and experimental simulations. The conclusion is that the proposed (six) modes are realistic but should be used in accordance to the period of the day. These conclusions should be applied in the future and a simulation has to be planned for a varied load.

## 11. Conclusions

A queue management system in an intelligent environment has been designed. This sensor-based system is context-aware and pro-active. It works according to the paradigm of pervasive and ubiquitous computing. Its main task is to recognise clients who appear in the monitored area, understand their behaviour, take into account the historical data related to objects and propose particular actions, that select the most convenient desk to exit the monitored area. Thus, it can cover both client support as well as simplification of the transaction finalisation when choosing the best store cash register.

We have proposed a multi-agent system, and its basic architecture, which allows for the rational dispersion of the system’s operational activities. The system meets the requirements for systems processing large amounts of sensory data, based on a sensor network, so as to get a context-aware system, which makes intelligent decisions in a smart city. The proposed contextual data taxonomy proved successful in this system. The formal properties of the proposed algorithms have been proven. The created simulation environment enables the testing of the basic principles of the proposed multi-agent system. Many simulation experiments have been carried out. The designed system, albeit in a prototype version, can be a good reference point for further system developments. The proposed system is feasible and viable. Another advantage of this system is revealed the current COVID-19 pandemic regulations, when keeping social distances and directing people with increased temperature to separate desks has become more important than before.

Further works for the system’s development should be carried with a special diligence in reference to the following non-functional system requirements: expansion, scalability, manageability, configurability, safety and stability.

## Figures and Tables

**Table 1:** Comparision for existing studies and solutions.

Authors and Articles	Field of Application and Description
Roy et al. [9], Pandey and Hanchate [10]	Car parking, avoid overloads, no context analysis and behaviour recognition
Yuan et al. [11]	Shopping markets, tags and sectors, profiles building, no context analysis
Berdaliyev and James [12]	Shopping markets, RFID technology, reducing queue delays, no context analysis and behaviour recognition
Stancu et al. [13]	General delivery service, experiments with a message broker, no context analysis and behaviour recognition
Lin and Lin [14]	Ticket dispenser in a restaurant, waiting time prediction, no context analysis and behaviour recognition

**Table 2:** The structure of basic messages transferred within the system. The meaning of particular elements is intuitive, whereby “timeStamp” means a time stamp, a certain moment in time, when biometric readings were performed. (Symbols for the table: “≡” is defined as; “=” is equal to; “+” conjunction; “[ ]” the choice of one possibility; “|” separator of disjoint choice related to [ ]; asterisks are for comments).

bioObs	≡	bio + timeStamp + iotId *biometric observation/data*
client	≡	obId + timeStamp + iotId + deskId *basic client data*
bio	≡	*registered biometric raw data*
timeStamp	≡	*date and time*
obId	≡	*client’s unique id*
iotId	≡	*iot’s unique id, where iot’area = [generalA|virtualA|queueA|exitA]*
deskId	≡	*desk’s unique id*

## Abbreviations

The following abbreviations are used in this manuscript:

ICT	Information and Communication Technology
IoT	Internet of Things
AmI	Ambient Intelligence
RFID	Radio-Frequency Identification
FIFO	First In First Out
BLE	Bluetooth Low Energy
MAS	Multi-agent systems
PDES	Parallel Discrete Event Simulation
